# Non-destructive Storage Time Prediction of Newhall Navel Oranges Based on the Characteristics of Rind Oil Glands

**DOI:** 10.3389/fpls.2022.811630

**Published:** 2022-03-29

**Authors:** Shumin Gao, Hanwen Kang, Xiaosong An, Yunjiang Cheng, Hong Chen, Yaohui Chen, Shanjun Li

**Affiliations:** ^1^College of Engineering, Huazhong Agricultural University, Wuhan, China; ^2^Key Laboratory of Agricultural Equipment in Mid-Lower Yangtze River, Ministry of Agriculture and Rural Affairs, Wuhan, China; ^3^College of Horticulture and Forestry Science, Huazhong Agricultural University, Wuhan, China; ^4^Department of Aerospace and Mechanical Engineering, Monash University, Clayton, VIC, Australia; ^5^National R&D Center for Citrus Preservation, Wuhan, China

**Keywords:** citrus fruit, storage time prediction, oil glands, non-destructive evaluation, deep learning

## Abstract

How to non-destructively and quickly estimate the storage time of citrus fruit is necessary and urgent for freshness control in the fruit market. As a feasibility study, we present a non-destructive method for storage time prediction of Newhall navel oranges by investigating the characteristics of the rind oil glands in this paper. Through the observation using a digital microscope, the oil glands were divided into three types and the change of their proportions could indicate the rind status as well as the storage time. Images of the rind of the oranges were taken in intervals of 10 days for 40 days, and they were used to train and test the proposed prediction models based on K-Nearest Neighbors (KNN) and deep learning algorithms, respectively. The KNN-based model demonstrated explicit features for storage time prediction based on the gland characteristics and reached a high accuracy of 93.0%, and the deep learning-based model attained an even higher accuracy of 96.0% due to its strong adaptability and robustness. The workflow presented can be readily replicated to develop non-destructive methods to predict the storage time of other types of citrus fruit with similar oil gland characteristics in different storage conditions featuring high efficiency and accuracy.

## Introduction

Citrus is an important agriculture commodity produced in over 140 countries, with the annual production of over 146 million tons (Liu et al., 2012). Due to the increase in cultivation area and improvement in management strategies (Díaz et al., 2017), the production of citrus fruit is expected to continuously increase in the future. Fresh citrus fruit usually go through commercial handling, transportation, wholesale or retail before finally reaching consumers; however, they may experience water loss and develop spoilage during postharvest storage, which will lessen their taste (Schirra et al., 2005; Marcilla et al., 2006). Currently, freshness evaluation is based on specially trained staff that assess the taste and aroma of the fruit samples (Guohua et al., 2012), which is a time-consuming and cost-expensive process featuring low repeatability and objectiveness. As freshness is strongly related to storage time under a specific storage condition (Mashabela et al., 2019; Tan et al., 2020), accurate and efficient methods for storage time prediction can help estimate freshness (Nesakumar et al., 2019), which can lead to better management in citrus shipping, storage, and retail procedures.

A common research path for fruit storage time prediction is to adopt volatile organic compounds (VOCs) gas sensors, whose non-destructive nature can be potentially related to practical applications. As every kind of fruit has a unique aroma made up of hundreds of VOCs, correspondence can usually be found among the flavor, product quality, storage time, and composition of VOCs (Baietto and Wilson, 2015; Liu et al., 2018b). One type of gas sensors adopts metal oxide semiconductors to detect the variations of the main components of VOCs, which have been used to predict the storage time of strawberries (Ghasemi-Varnamkhasti et al., 2019) and peaches (Liu et al., 2018a). To improve the detection sensitivity and humidity tolerance, quartz crystal microbalance (QCM) sensors are proposed for the estimation of fruit storage time, maturity, and shelf life (Nimsuk and Nakamoto, 2008; Adak et al., 2017). However, these gas sensors are rarely applied to citrus fruit, which might be caused by the subtle changes in their VOCs contents that are difficult to measure and differentiate (Cui et al., 2016). In a recent study, Raman spectroscopy is used to relate the intensity of rind carotenoid signals to the storage time (Nekvapil et al., 2018). Despite that it might be a useful solution for citrus freshness control, the signal quality depends on the instrument and user capability, making it hard to develop a well-accepted standard for practical *in-situ* applications.

As the external appearance of the fruit is the most important criterion for customers to evaluate the storage time, potential correlation between the rind status and storage time is worthy exploring. The peel turgor is an essential parameter of rind quality and the loss of turgor pressure is probably the main factor of rind compaction, as well as the decrease of attraction to customers (Alferez et al., 2010). However, non-destructive methods to quantify this process for storage time prediction still remain unavailable. On the other hand, secretory cavities occur naturally in all species of the family Rutaceae, and in the genus Citrus they are often referred to as oil glands. They can be found in the stem, mesophyll of leaves, all parts of the flower except the stamens, and the exocarp layer of the fruit rind, in the center of which an essential oil-accumulating reservoir develops (Turner, 1999; Knight et al., 2001). The essential oil is important in the protection of the plants due to their bactericidal and fungicidal nature, and their strong odor may also attract some insects to favor the dispersion of seeds and pollens (Rodov et al., 1995; Palazzolo et al., 2013). While it is still contentious whether the central cavity forms by lysigeny or schizogeny (Turner et al., 1998), the enlargement of oil glands has been anatomically observed and investigated in a series of studies (Liang et al., 2006), indicating that the initialization of glands is restricted to the early stages of fruit development while the enlargement is up to fruit maturity (Bennici and Tani, 2004; Hou et al., 2019). As a result, the total gland number is quite constant for mature fruit. Moreover, according to Bosabalidis and Tsekos (1982), glands are attached to the fruit epidermis by a stalk-like structure, which tends to become less obvious when the fruit develops. For mature citrus fruit, the gland stalk is even reduced to only a few cell layers in depth below the epidermis, making the oil glands outstanding on the appearance of the rind. In one study, gland characteristics of citrus fruit, such as gland size and density, have been investigated for maturity assessment, reaching a correlation coefficient of 0.77 (Hongwiangjan et al., 2015). Although little research has been conducted to reveal the evolution of oil glands during postharvest storage, the oil within the glands generally decreases during storage due to water loss, which is also the key factor of the loss of peel turgor. Ghanem et al. (2012) conducted a dehydration test, whose result further demonstrates that the compaction of the citrus peel is in accordance with the shrinkage of oil glands due to evaporated water. This characteristic might indicate the rind status in an easily quantifiable manner, which can be potentially adopted to develop non-destructive methods for storage time prediction.

This study aims to prospect the relationship, if any, among the characteristics of the rind oil glands, rind status, and storage time, based on which model-based prediction methods can be developed for non-destructive storage time prediction. The predicted storage time can be then used as a key parameter for freshness evaluation. As a feasibility study, we investigated the evolution of the rind oil glands of Newhall navel oranges in intervals of 10 days for continuous 40 days using a digital microscope, and K-Nearest Neighbors (KNN) and deep learning algorithms, respectively, were adopted to analyze the high-resolution images and develop two types of prediction models. The workflow presented can be readily replicated to develop non-destructive methods to predict the storage time of other types of citrus fruit with similar oil gland characteristics in different storage conditions featuring high efficiency and accuracy.

## Materials and Methods

### Sample Fruit

Sample Newhall navel oranges (Citrus synesis) were harvested at a commercial orchid in Zigui, Hubei Province, China (111.0°N, 30.8°E) in December 2019. This type of oranges can usually be stored under room temperature for up to 40 days before their external appearance turn observably unfavorable, but within 40 days the changes in their appearance are hard to distinguish. The fruit were first cleaned on a citrus processing line and no waxing was applied, and they were then transported to Wuhan, China *via* air flight on the same day. As we focus mainly on the storage time prediction of healthy oranges in this study, we manually inspected the oranges and selected 600 ones with a sound surface. These fruits were then stored in a ventilated chamber with the environment similar to the warehouse (approx.10°C, 65%RH, no natural light) for a storage period of 40 days.

### Chemical and Mechanical Measurement

Four hundred and fifty oranges in total were chosen for chemical and mechanical measurements in this study, in which the pH value, sugar-acid ratio, hardness, and weight were assessed. These oranges were evenly divided into five groups at random, and one group was adopted for the measurements on day 0 (denoted as the beginning of the test), 10, 20, 30, and 40, respectively.

The measurement of the pH value was conducted on 30 sample oranges each time. A small fruit sample was cut from each orange and then squeezed with double-layer gauze at room temperature, and an automated pH meter (SevenExcellence, METTLER TOLEDO) was used to assess the pH value of the juice. This measurement was replicated three times and the average was recorded. The sugar-acid ratio was obtained using a refractometer (ATGO PAL-BX/ACID1) following the same process on another 30 oranges. The remaining 30 oranges were first adopted for weight measurement, and the puncture test was then carried out on their equatorial region using a portable fruit hardness tester (Gy2) for hardness measurement.

### Microscope Image Acquisition

A high-resolution digital microscope (VHX-6000) was adopted to observe the rind oil glands of 150 oranges during postharvest storage. Due to its capacity to obtain the depth information with high accuracy, it can well capture the evolutionary characteristics of the rind oil glands. Image acquisition was conducted in a chamber with no natural light. Since the light was only provided by the microscope itself, the same light condition was ensured all through the observation. According to, oil glands are generally uniformly distributed at the equatorial region of the rind of citrus fruit, and we therefore took one image every 10 days under 50× magnification randomly at the equatorial region for each orange, resulting in 150 images collected each time.

### Model-Based Prediction Methods

**Figure 1:**
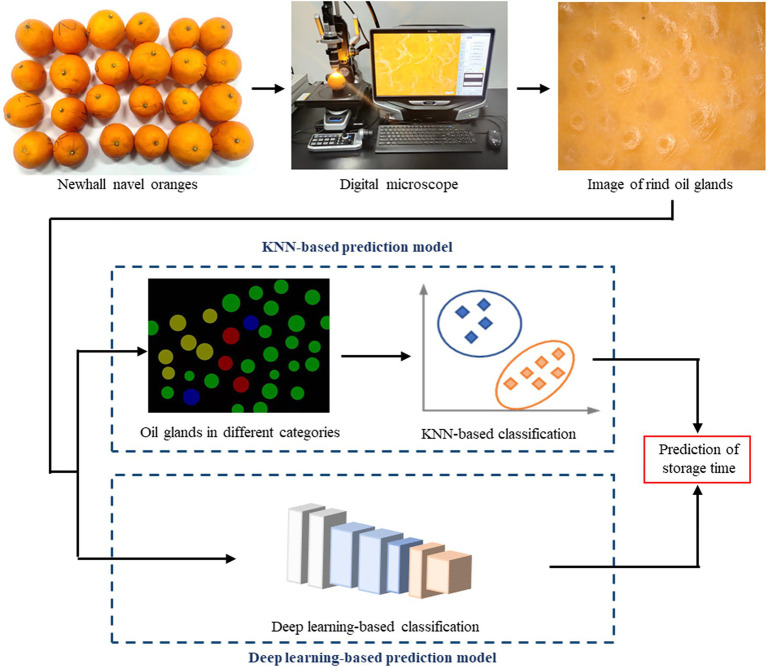
Flowchart of the development of the K-Nearest Neighbors (KNN) and deep learning-based prediction models for the storage time of the Newhall oranges.

Two prediction models based on KNN and deep learning algorithms, respectively, were developed for the prediction of the storage time, as shown in Figure 1. The KNN-based prediction model explicitly demonstrated the classification criteria and provided insights to relate the evolution of the oil glands to the rind status and storage time. The deep learning-based prediction model was capable of learning the features by itself, which was anticipated to achieve better performance in prediction accuracy and robustness.

### KNN-Based Prediction Model

K-Nearest Neighbors is one of the most commonly used unsupervised-learning method in machine learning for classification, which can be adopted for storage time prediction as the images were categorized into five classes based on the collection date. The algorithm first calculates the distance (Manhattan or Euclidean distance) between the unknown sample and K-nearest known samples, and it then classifies the unknown sample based on the distance and classes of the K-nearest samples (Chen et al., 2017). In this work, the oil glands captured in each image were identified into three types based on their evolutionary characteristics, which will be discussed in detail in Section “Evolution of rind oil glands,” and the features to perform storage time prediction were the proportions of different glands. For the images obtained each time, 130 were randomly selected as the training set and the remaining 20 were used as the test set.

### Deep Learning-Based Prediction Model

Convolution Neural Network (CNN) achieves superior performance in computer vision tasks such as classification (Chen et al., 2021), object detection (Kang and Chen, 2020a), and segmentation (Kang and Chen, 2020b). Here, we applied a 50-layer CNN model Residual-Network (ResNet-50) to directly predict the storage time of the oranges based on the images obtained, which was also treated as a classification task. ResNet-50 applied the residual convolution module, in which a shortcut connection was introduced between the input and output, to improve the accuracy and trainability of the network. The model was then trained to predict the storage time of the oranges into one of the five classes using the same training set of the KNN-based model. The Multiple Level Perception (MLP) layers of the ResNet-50 model were redesigned to fit our designed output. The global-pooling layer of the block of the original ResNet-50 outputted a feature map with the size of 1 × 1 × 2048, and the generated feature map was reshaped into a feature vector (2048 × 1). After that, two fully-connected layers, of which the sizes were 256 and 6, respectively, were used to generate the prediction of the storage time. Batch-norm and drop-out were adopted after each fully connected layer to improve the performance of the network model.

## Results

### Chemical and Mechanical Analysis

**Figure 2:**
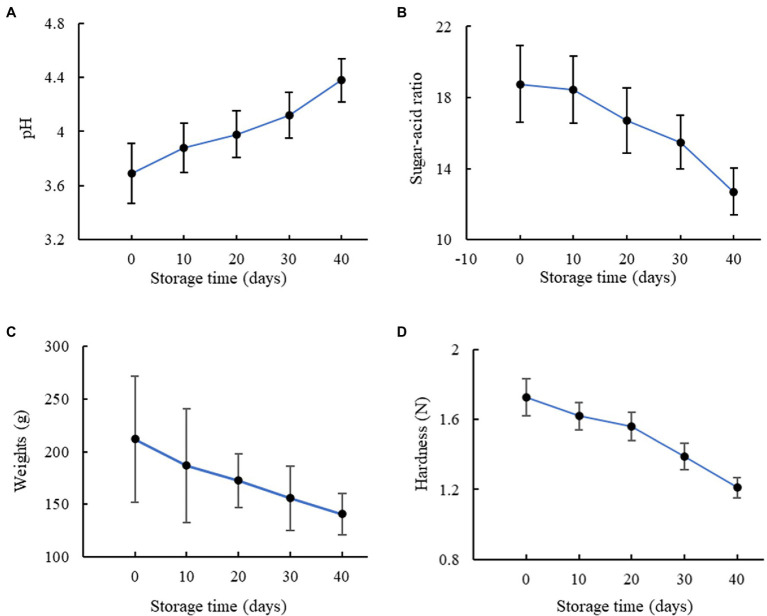
Results of the measurement of the **(A)** pH value, **(B)** sugar-acid ratio, **(C)** weight, and **(D)** hardness.

The pH value, sugar-acid ratio, weight, and hardness of these oranges in different storage periods were obtained through chemical and mechanical experiments. It can be observed from Figure 2A that the average pH value of the oranges increased with increasing storage time, which was mainly due to the degradation of the ascorbic acid (Touati et al., 2016). For the oranges freshly collected on Day 0, their juice had a relatively high acidic level with an average pH value of 3.6. While the juice was still slightly acidic on Day 40, the average pH value obtained was 20.0% higher than that of the first day. The result of the sugar-acid ratio is presented in Figure 2B, showing a generally decreasing trend during the storage period. For Day 0 and 10, the average sugar-acid ratios of the oranges were 18.7 and 18.3, respectively, indicating a satisfactorily sweet taste. The sugar and acid contents deteriorated due to respiration during storage, and the experimental data indicates a higher decreasing speed of sugar concentration than that of the acid during storage, resulting in a decrease in the sugar-acid ratio. However, these indexes were associated with significant variances, based on which accurate prediction of the storage time was difficult to perform. Moreover, their destructive nature also constrains their potential applications. According to Figure 2C, the weights of the oranges decreased with increasing storage time, which can be related to respiration and loss of water, but the variances of the results were even more significant. Figure 2D shows the result of the hardness test. A generally linear negative correlation was found between the hardness and storage time, which was mainly induced by the loss of water as well (Rivera et al., 2021). As the variances of the experimental data were relatively small, the hardness might be useful to distinguish the oranges into different storage periods. However, devices to non-destructively measure the hardness of citrus fruit are not commercially available at the moment. As a result, although the pH value, sugar-acid ratio, weight, and hardness demonstrated the decreased fruit quality with increasing storage time, they are not ideal indexes for non-destructive storage time prediction.

### Evolution of Rind Oil Glands

**Figure 3:**
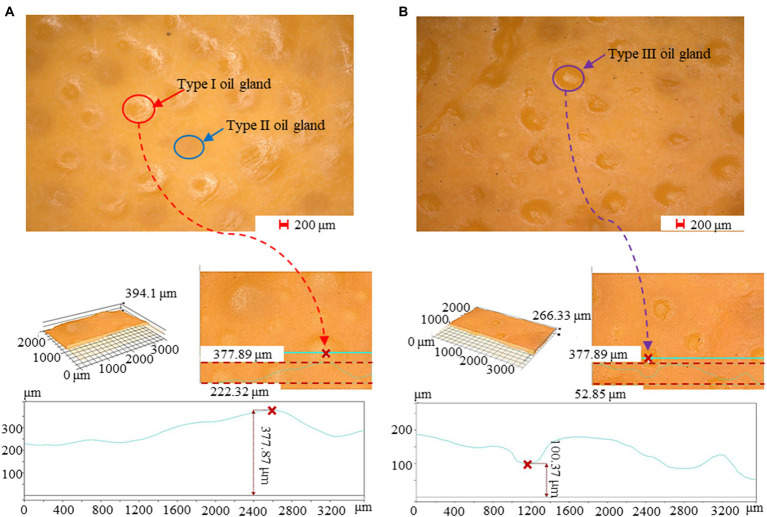
Microscope observation of the oil glands. **(A)** The characteristics of the oil glands on the 0th day, in which the oil glands were filled up essential oil and had a convex surface. **(B)** The characteristics of the oil glands on the 40th day, in which the essential oil was limited and the gland surface was concave.

The evolution of the rind oil glands during postharvest storage was analyzed based on the obtained microscope images. For the freshly-collected oranges shown in Figure 3A, oil glands were the most prominent characteristics on the orange rind under 50×-magnification observation. Due to the accumulation of essential oil, most of the oil glands presented a convex surface with a light contrast in color comparing with other parts of the rind, and these convex surfaces can be further confirmed with the depth information. Moreover, there were also several oil glands with a flat surface, which might be due to the slow accumulation of essential oil during fruit development or slight dissipation when the fruit was mature. According to, the essential oil content in citrus fruit increases promptly before the mature stage and then drops slowly as a result of the elimination of oil. For the oranges preserved for 40 days, as shown in Figure 3B, most of the oil glands experienced a significant decline in the oil content, making their surfaces concave appearing on the rind.

**Figure 4:**
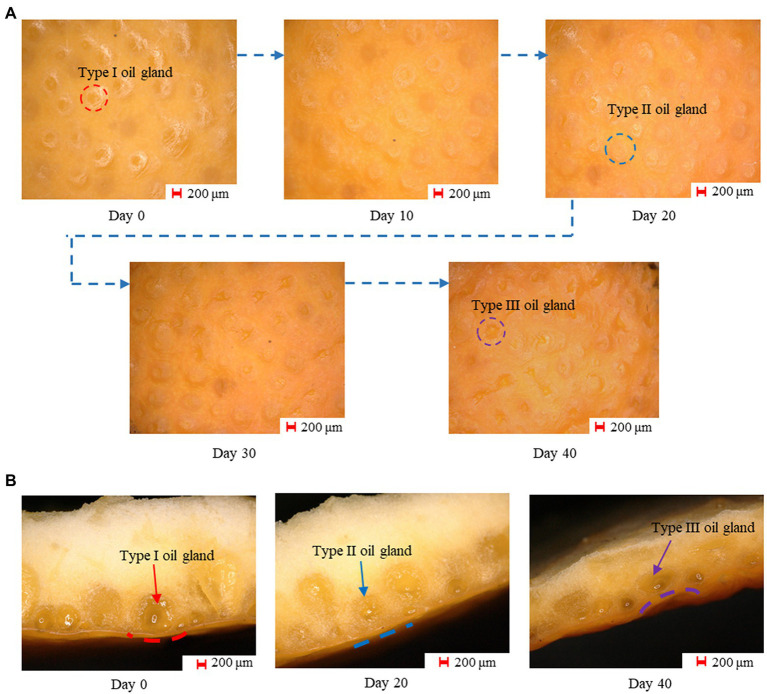
**(A)** The images captured in different storage periods. An increasing number of Type I oil glands turned into Type II and III glands with increasing storage time, and most of the glands were Type III glands on day 40. **(B)** The cross-section of the rinds on day 0, 20, and 40, on which different types of oil glands can be observed.

To quantify the characteristics of the oil glands at different storage periods, we classified them into three types in a relatively simple way. Type I oil glands referred to those filled with essential oil and have convex surfaces observed from the rind, and Type II glands were characterized by a flat surface when they experienced the decrease in oil content. For the oranges stored for a relatively long time, little essential oil was left and the gland surfaces were obviously concave and in a darker color, and they were therefore classified as Type III glands. Figure 4A shows the images of the rind from Day 0 to Day 40, and it can be found that although Type I glands dominated the rind at the beginning, they gradually turned into Type II and Type III glands with increasing storage time. On Day 40, most of the glands were characterized as Type III glands, which can hardly be observed in freshly harvested oranges. Figure 4B compares the cross-section of the rinds on Day 0, 20, and 40, which further confirms that the elimination of oil was the key factor that turned the glands flat or even concave. Moreover, the rind itself also experienced the loss of water during postharvest storage and became thinner, which would decrease the rind turgor and result in the rind compaction (Rivera et al., 2021). As this process is in conjunction with the evolution of the oil glands, the gland characteristics can be used as an indicator of the rind status that can be easily quantified, which will be discussed in detail in “Modeling and Analysis.” Compared with other indexes to indicate the rind status such as the rind turgor and water potential, the proposed new indicator can be obtained immediately using images in a non-destructive manner, which can be conveniently adopted in real-world applications for storage time prediction.

## Modeling and Analysis

### Evaluation Metrics

We used accuracy to evaluate the performance of the KNN and deep learning-based prediction models, respectively. Accuracy is formulated as


(1) $$Accuracy=\frac{1}{n}\overset{n}{\underset{i=1}{\sum }}I\left(f\left(X_{i}\right)=Y_{i}\right)$$

where n is the total number of samples in the test set, $f\left(X_{i}\right)$ is the predicted classes of the $i_{th}$ sample by the model, $Y_{i}$ is the ground truth label of the $i_{th}$ sample, and *I* is the function to determine whether $f\left(X_{i}\right)$ equals $Y_{i}$.

### KNN-Based Modeling

**Figure 5:**
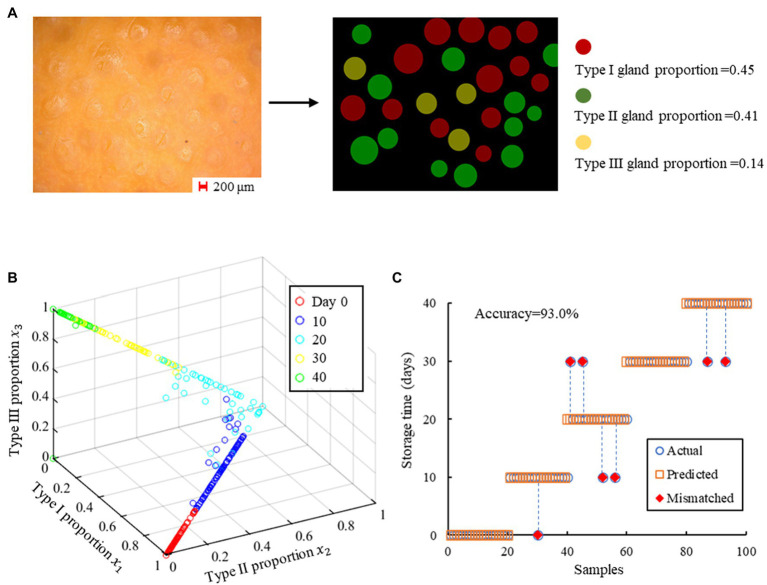
The results of the KNN-based prediction model. **(A)** The procedure to obtain the proportions of different glands from an image. **(B)** The training set of the KNN-based prediction model, in which the proportions of three types of oil glands in each picture are the features to perform freshness prediction. **(C)** Comparison between the predicted and actual storage time.

According to Section “Evolution of Rind Oil Glands,” the total number of the oil glands remained the same for the oranges during postharvest storage, while the oil content decreased with increasing storage time. As a result, the proportion of Type I glands would gradually decrease, and the proportion of Type II glands would first increase and then decrease as the glands would further turn into Type III glands. While the individual difference of the gland number is obvious, the proportions of three types of glands for each orange at different storage period were quite consistent. Here, we adopted the proportions of three types of glands in the images as the features for KNN-based classification, as shown in Figure 5A. Figure 5B shows the distribution of the training set, in which the data from the same storage time gathered spatially, demonstrating the efficacy of the selected features.

Euclidean distance was adopted for the criteria of classification, and the total distance *D* of the test sample and its K-nearest neighbors was therefore formulated as


(2) $$D=\overset{k}{\underset{m=1}{\sum }}\sqrt{\overset{3}{\underset{i=1}{\sum }}{\left(x_{m,i}-y_{m,i}\right)}^{2}}$$

where $x_{m,i}$ is the proportions of different glands of the test sample, and $y_{m,i}$ is the proportions of different glands of a neighbor sample from the training set. The test sample was then classified into the category, where the majority of its K-nearest neighbors belonged to.

We conducted the computation using the scikit-learn machine learning library (version 0.19.0) in Python. In order to achieve better model performance, the parameters of the model were debugged based on the approach presented in Qiu et al. (2008). Different numbers of neighbors were tested for comparison and the results are presented in Table 1. The highest accuracy achieved is 93.0% with five nearest neighbors, with the parameters of the model set as n_neighbors = 5, weights = uniform, leaf_size = 30, metric=“minkowski,” and n_jobs = 1. These parameters were then adopted to predict the storage time of the test samples, and the comparison between the predicted and actual storage time is presented in Figure 5C. There are only seven out of 100 test samples misclassified, and the errors are all within 10 days, demonstrating the feasibility to use oil gland characteristics and a KNN-based model to predict the storage time of the Newhall navel oranges.

**Table 1:** Accuracy of the KNN-based prediction model using different number of neighbors.

Number of neighbors	Accuracy (%)
1	92.0
3	92.0
5	93.0
7	91.0
9	92.0

### Deep Learning-Based Modeling

**Figure 6:**
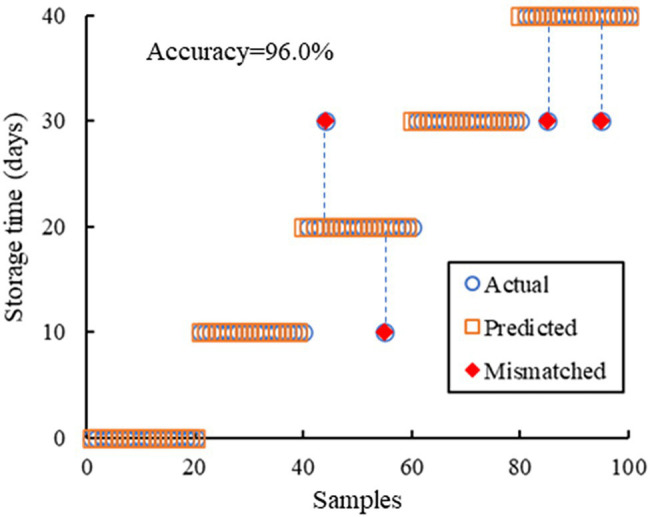
The results of the predicted storage time of the deep learning-based model, which are compared with the actual storage time.

The deep learning-based model can directly predict the storage time of the oranges based on the microscopy images. The implementation code of the original ResNet-50 model was from open-source code in Github, which was programmed by using the slim library in TensorFlow 1.15. To achieve better accuracy on storage time prediction, the MLP layers of the model was redesigned to fit outputs. We trained ResNet-50 by using the Adam-optimizer, and the pre-trained weights of the convolutional layers of the ResNet-50 model were frozen. As a result, only the weights in the MLP layers were trained. During the training process, the network was trained with 50 epochs and the learning rate was 0.001. Image augmentation methods including flip, rotation, clip, and color adjustment in HSV color space were introduced. The results of storage time prediction are presented in Table 2. The overall accuracy of the trained ResNet-50 model on storage time prediction is 96.0%, with only four mismatched out of 100 test samples and the errors all within 10 days, as shown in Figure 6.

**Table 2:** Accuracy of the deep learning-based prediction model.

Storage time (days)	Accuracy (%)
0	100
10	100
20	90
30	100
40	96
Overall	96

## Discussion

Research on citrus storage time prediction is limited as the changes of their VOCs contents are difficult to differentiate *via* gas sensors (Cui et al., 2016). In one study, a freshness coefficient of citrus fruit was proposed based on the Raman intensity of rind carotenoids, which would decrease with increasing storage time (Nekvapil et al., 2018). However, this coefficient has high variances which might influence the prediction accuracy, and the intensity of Raman signals also relies on the specific equipment adopted. These issues constrain the application of practical methods to predict the storage time of citrus fruit. This paper reveals the evolutionary characteristics of the Newhall navel oranges during postharvest storage, and a feasibility study is presented to relate these characteristics to the rind status and storage time. One obvious advantage of the proposed method is the high objectiveness as the captured gland characteristics are not likely to be influenced by different equipment for image acquisition. The non-destructive nature is also appealing to the citrus fruit market, and *in situ* applications can be developed if a portable microscope is used.

**Figure 7:**
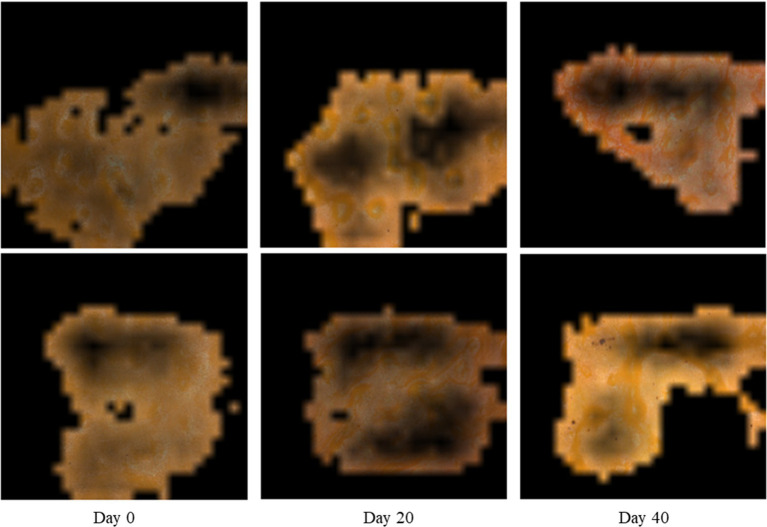
Feature visualization results of the deep learning-based model in different storage time.

The KNN and deep learning-based prediction models both achieved high prediction accuracy with the test samples, with the errors all within 10 days. The KNN-based model presented explicit criteria for classification, which also provided insights for the evolution of the oil glands during postharvest storage. To explore potential classification criteria of the deep learning-based model, we visualized the weighted sum of the feature maps in the last convolution layer by multiplying the feature maps with the corresponding weights in the MLP layers. That is, the feature maps with higher weights in the MLP layers would be highlighted. Then, to investigate the weights of different features that the classification network relied on for storage time prediction, we upsampled the weighted sum feature maps and multiplied them with the input image. As shown in Figure 7, although the oil glands gathered densely in some part of the images and distributed rather loosely in other parts, the highlighted areas by the network included a large amount of glands, indicating that the glands have higher weights in classification. This demonstrates that gland characteristics are also important for the deep learning-based model.

One issue for the presented work is that we focused only on Newhall navel oranges stored at a specific storage condition, and the evolution of the oil glands might be different for different citrus species and storage conditions. However, this study aims to evaluate the feasibility to correlate the evolutionary characteristics of the rind oil glands with the rind status, through which the storage time can be predicted non-destructively, and specific storage time prediction models for different citrus species and storage conditions are therefore out of the scope. The comparison between the predicted and actual storage time based on the sample images achieved high prediction accuracy, demonstrating the potential of the proposed method. Although this method might not apply to citrus species whose oil glands are difficult to observe or with a concave surface when freshly harvested, the workflow presented can be readily replicated to develop new storage time prediction models for other citrus species with similar oil gland characteristics to Newhall navel oranges under different storage conditions. We will also include more citrus species and storage conditions in our future work to further evaluate the proposed method.

## Conclusion

In this paper, the feasibility of performing storage time prediction of Newhall navel oranges based on the evolutionary characteristics of the rind oil glands has been evaluated, and two prediction models based on KNN and deep learning algorithms, respectively, have been proposed. The observation through microscope images demonstrated that the surfaces of the rind oil glands would turn from convex to concave due to the elimination of essential oil during postharvest storage, which is in conjunction with the process of the decrease of rind turgor and can be related to the rind status. The KNN-based model adopted the proportions of different types of oil glands as the features for classification, reaching a high prediction accuracy of 93.0%. The deep learning-based model directly predicted the storage time according to the images, and a higher accuracy of 96.0% was also achieved. Moreover, the prediction errors of both models were all within 10 days. The workflow presented can be readily replicated to develop storage time prediction tools for various citrus fruit with similar gland characteristics to Newhall navel oranges under different storage conditions.

## Data Availability Statement

The raw data supporting the conclusions of this article will be made available by the authors, without undue reservation.

## Author Contributions

YC, SL, and HC contributed to conception and design of the study. SG organized the database and wrote the first draft of the manuscript. SG and HK performed the statistical analysis. All authors contributed to the article and approved the submitted version.

## Funding

This research was funded by National Key R&D Program (2020YFD1000101) and Special Funds for the Construction of Industrial Technology System of Modern Agriculture (Citrus; CARS-26), Construction Project of Citrus Whole Course Mechanized Scientific Research Base [Agricultural Development Facility 297 (2017) 19], and Hubei Agricultural Science and Technology Innovation Action Project.

## Conflict of Interest

The authors declare that the research was conducted in the absence of any commercial or financial relationships that could be construed as a potential conflict of interest.

## Publisher’s Note

All claims expressed in this article are solely those of the authors and do not necessarily represent those of their affiliated organizations, or those of the publisher, the editors and the reviewers. Any product that may be evaluated in this article, or claim that may be made by its manufacturer, is not guaranteed or endorsed by the publisher.
